# Qualitative Differences in Capsidless L-Particles Released as a By-Product of Bovine Herpesvirus 1 and Herpes Simplex Virus 1 Infections

**DOI:** 10.1128/JVI.01259-18

**Published:** 2018-10-29

**Authors:** Tiffany Russell, Ben Bleasdale, Michael Hollinshead, Gillian Elliott

**Affiliations:** a Department of Microbial Sciences, Faculty of Health and Medical Sciences, University of Surrey, Guildford, United Kingdom; b Section of Virology, Imperial College London, London, United Kingdom; Northwestern University

**Keywords:** BoHV-1, envelopment, HSV-1, L-particles, morphogenesis, tegument

## Abstract

Despite differences in the pathogenesis and host range of alphaherpesviruses, many stages of their morphogenesis are thought to be conserved. Here, an ultrastructural study of bovine herpesvirus 1 (BoHV-1) envelopment revealed profiles similar to those previously found for herpes simplex virus 1 (HSV-1), with BoHV-1 capsids associating with endocytic tubules. Consistent with the similarity of their genomes and envelopment strategies, the proteomic compositions of BoHV-1 and HSV-1 virions were also comparable. However, BoHV-1 morphogenesis exhibited a diversity in envelopment events. First, heterogeneous primary envelopment profiles were readily detectable at the inner nuclear membrane of BoHV-1-infected cells. Second, the BoHV-1 progeny comprised not just full virions but also an abundance of capsidless, noninfectious light particles (L-particles) that were released from the infected cells in numbers similar to those of virions and in the absence of DNA replication. Proteomic analysis of BoHV-1 L-particles and the much less abundant HSV-1 L-particles revealed that they contained the same complement of envelope proteins as virions but showed variations in tegument content. In the case of HSV-1, the U_L_46 tegument protein was reproducibly found to be >6-fold enriched in HSV-1 L-particles. More strikingly, the tegument proteins U_L_36, U_L_37, U_L_21, and U_L_16 were depleted in BoHV-1 but not HSV-1 L-particles. We propose that these combined differences reflect the presence of truly segregated “inner” and “outer” teguments in BoHV-1, making it a critical system for studying the structure and process of tegumentation and envelopment.

**IMPORTANCE** The alphaherpesvirus family includes viruses that infect humans and animals. Hence, not only do they have a significant impact on human health, but they also have a substantial economic impact on the farming industry. While the pathogenic manifestations of the individual viruses differ from host to host, their relative genetic compositions suggest similarity at the molecular level. This study provides a side-by-side comparison of the particle outputs from the major human pathogen HSV-1 and the veterinary pathogen BoHV-1. Ultrastructural and proteomic analyses have revealed that both viruses have broadly similar morphogenesis profiles and infectious virus compositions. However, the demonstration that BoHV-1 has the capacity to generate vast numbers of capsidless enveloped particles that differ from those produced by HSV-1 in composition implies a divergence in the cell biology of these viruses that impacts our general understanding of alphaherpesvirus morphogenesis.

## Abstract (precis)

The alphaherpesvirus family includes viruses that infect humans and animals. Hence, not only do they have a significant impact on human health, but they also have a substantial economic impact on the farming industry. While the pathogenic manifestations of the individual viruses differ from host to host, their relative genetic compositions suggest similarity at the molecular level. This study provides a side-by-side comparison of the particle outputs from the major human pathogen HSV-1 and the veterinary pathogen BoHV-1. Ultrastructural and proteomic analyses have revealed that both viruses have broadly similar morphogenesis profiles and infectious virus compositions. However, the demonstration that BoHV-1 has the capacity to generate vast numbers of capsidless enveloped particles that differ from those produced by HSV-1 in composition implies a divergence in the cell biology of these viruses that impacts our general understanding of alphaherpesvirus morphogenesis.

## INTRODUCTION

The alphaherpesvirus subfamily comprises a group of complex viruses that includes important human and animal pathogens, such as herpes simplex virus 1 (HSV-1) and bovine herpesvirus 1 (BoHV-1). Such viruses have a significant impact on human or animal health (1, 2) and in the case of agricultural infections can have a substantial economic impact (3, 4). Despite these viruses showing a diverse range of clinical manifestations (5, 6), they show conservation in genome composition and virion morphology (7, 8). The alphaherpesvirus particle consists of the DNA-containing capsid surrounded by a proteinaceous layer known as the tegument, comprised of over 20 virus-encoded proteins and multiple cell proteins, which links the capsid with its host membrane-derived envelope containing multiple virus-encoded glycoproteins (9, 10). While studies on virion assembly have tended to center around HSV-1 and pseudorabies virus (PRV), there is growing interest in utilizing other alphaherpesviruses to enhance our understanding of specific morphogenesis details that are still missing.

The currently favored model for assembly of the complex virion is termed the envelopment-deenvelopment-reenvelopment model, in which capsids form in the nucleus and bud through the inner nuclear membrane (INM) as a primary virion (11–13). The virus-encoded machinery required for this envelopment, termed the nuclear egress complex or NEC, consists of U_L_31 and U_L_34, considered to be the tegument and envelope of the primary virion (14). This machinery is autonomous and can function when expressed in isolation or even *in vitro* (15, 16). The primary envelope is lost by fusion with the outer nuclear membrane (ONM), releasing naked capsids into the cytosol (11, 12). This cytoplasmic capsid is subsequently enveloped in cellular membranes together with the complement of tegument proteins to form the mature virion.

The cellular location of alphaherpesvirus secondary envelopment has been a point of contention for many years. For HSV-1 at least, in a model derived from ultrastructural and Rab GTPase depletion studies, we have proposed that clathrin-mediated endocytosis of tubules from the plasma membrane provides the main source of the HSV-1 envelope, with a concomitant cycling of virus envelope proteins through the plasma membrane to the endocytic wrapping tubules (17). Virus egress would then result from the natural recycling of these membranes to the cell surface. This model is in agreement with previous studies from others (18) and has been supported by more recent studies showing that glycoproteins must be transported to the plasma membrane prior to envelopment taking place (19, 20).

One idiosyncratic feature of the alphaherpesvirus envelopment pathway that has not been fully explored for understanding the molecular mechanisms involved in envelopment is the production of noninfectious light particles (L-particles) that lack the viral DNA-containing capsid but contain an enveloped tegument structure (21–26). These L-particles could also help in understanding the process of tegumentation, i.e., where and when the numerous tegument proteins are recruited to the assembling virion. Combinations of genetic and protein-protein interaction studies have led to the concept of inner and outer tegument proteins, with inner tegument proteins (such as U_L_36 and U_L_37) linking the capsid to the tegument and outer tegument proteins (such as U_L_49) linking the tegument to the envelope (27). Inner tegument proteins would hence be assembled onto the capsid at any point prior to envelopment, with some evidence suggesting that U_L_36 may already be present on intranuclear capsids (28, 29). While outer tegument proteins are proposed to be recruited to the envelope by interactions with the cytoplasmic tails of glycoproteins, conclusive evidence for such recruitment is still limited, with only two examples, U_L_11 and U_L_49, so far definitively shown to be assembled in this way (30–33). This issue is further compounded by the fact that many of the tegument proteins, even the major ones such as U_L_47 and U_L_49, which are considered to be structurally important, are dispensable for virus growth and therefore not required for virion formation (34–37). The assembly of tegument proteins into L-particles may offer a different route to understanding the molecular interactions that occur during alphaherpesvirus envelopment.

In this study, we employed a dual approach based on ultrastructural studies of morphogenesis and virion proteomics to carry out comparative studies of HSV-1 and BoHV-1 morphogenesis. We demonstrate that BoHV-1 assembly utilizes recently endocytosed material for envelopment in a manner analogous to that of HSV-1, while side-by-side proteomic analysis also confirmed similar compositions for HSV-1 and BoHV-1 virions. BoHV-1 was seen to produce large numbers of capsidless L-particles that were released in equivalent numbers to infectious virions. We report the first proteomic analysis of alphaherpesvirus L-particles indicating that the BoHV-1 but not the HSV-1 L-particles were depleted for proteins that are commonly classified as part of the inner tegument. These results reveal differences in the particle outputs from these two viruses that make BoHV-1 L-particles a useful model with which to study tegumentation and envelopment.

## RESULTS

### BoHV-1 capsids are wrapped in endocytic membranes.

**FIG 1:**
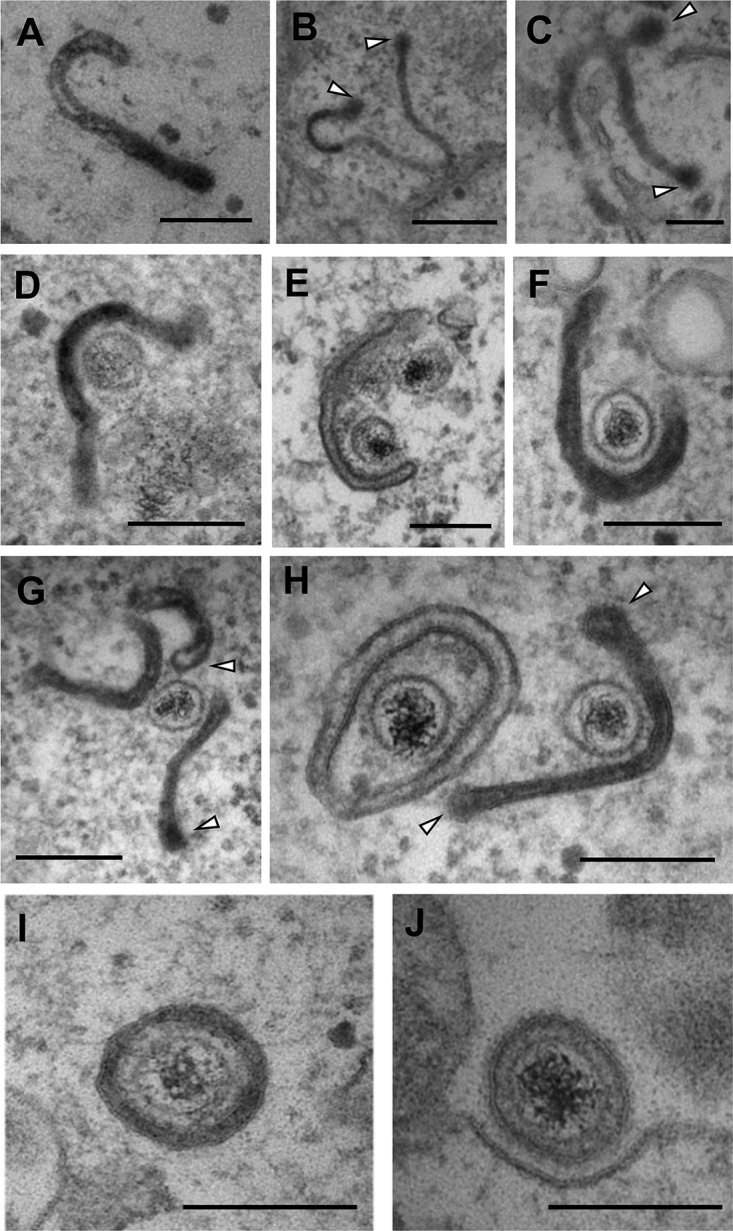
BoHV-1 capsids are wrapped in tubular endocytic membranes. MDBK cells were infected at a multiplicity of infection (MOI) of 5 with BoHV-1, and HRP was added to the medium for 30 min at 12 h postinfection (hpi). Samples were fixed, processed, and imaged by transmission electron microscopy. White arrows in panels B, C, G, and H indicate the budded terminal domains of endocytic tubules. Bars = 500 nm (B) and 200 nm (A and C to J).

We have previously shown that HSV-1 virions acquire their final envelopes from glycoprotein-containing endocytic tubular membranes that have been recently retrieved from the cytoplasmic membrane, by using horseradish peroxide (HRP) to label fluid-phase endocytic events in infected cells (17). To investigate BoHV-1 envelopment, we therefore carried out the same labeling of bovine MDBK cells, one of the few cell types in which BoHV-1 replicates, 12 h after infection at a multiplicity of 2. These studies revealed the presence of many HRP-labeled endocytic tubules with a curved profile in the cytoplasm (Fig. 1A to C). These endocytic tubules frequently carried a budded terminal domain (Fig. 1B, arrowheads), which had an indistinct peripheral coating when observed at higher magnification (Fig. 1C, arrowheads), consistent with the appearance of clathrin coats, as we have observed previously (17). Virus capsids were frequently found in tight association with these endocytic tubules in the cytoplasm of the infected cells (Fig. 1D to H). Furthermore, many tubules that were associated with capsids retained the budded terminal domain, consistent with a clathrin coat (Fig. 1G and H, arrowheads). Fully enveloped virions wrapped in double membranes were detected in the cytoplasm (Fig. 1I), while particles enclosed in a single membrane were observed outside the cell, consistent with our previous HSV-1 studies (Fig. 1J).

### Comparative proteomic analysis of extracellular BoHV-1 and HSV-1 virions.

**FIG 2:**
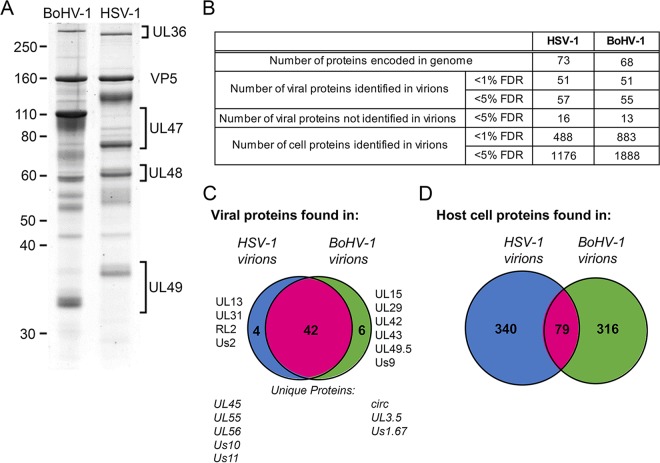
Purification and proteomic characterization of extracellular HSV-1 and BoHV-1 virions. Confluent monolayers of HaCaT cells and MDBK cells were infected with HSV-1 and BoHV-1, respectively; at full cytopathic effect, cells were harvested; and virions were isolated by separation on a 5 to 15% Ficoll gradient. (A) BoHV-1 and HSV-1 virions were separated by 10% SDS-PAGE and stained with Coomassie blue. Size markers are shown in kilodaltons. (B) Summary of the numbers of proteins identified in HSV-1 and BoHV-1 virions, at <5% and <1% FDRs. (C and D) Venn diagrams showing the overlap in virus proteins (C) and host cell proteins (D) identified in HSV-1 and BoHV-1 virions.

We next determined the relative protein contents of HSV-1 and BoHV-1 virions by isolating extracellular virions from infected cultures of physiologically relevant cells, namely, the HaCaT human keratinocyte cell line and the bovine epithelial MDBK cell line, respectively. The virions were banded on a Ficoll gradient, and approximately equivalent numbers of virions were solubilized and separated by SDS-PAGE, followed by staining with Coomassie blue to confirm the presence and purity of virions (Fig. 2A). Based on their characteristic sizes and previous studies, several key components of virions were identified, including the major capsid protein (VP5) and the tegument proteins U_L_36, U_L_47, U_L_48, and U_L_49. Of note, all these proteins, with the exception of U_L_47, were present at equivalent levels in both types of virions (Fig. 2A). In the case of U_L_47, in BoHV-1, this protein (also known as VP8) is known to be packaged in unusually large amounts (38). The BoHV-1 and HSV-1 virion proteins were subsequently separated by SDS-PAGE and subjected to in-gel tryptic digestion, followed by fractionation using a nanoscale high-performance liquid chromatography (nano-HPLC) system and tandem mass spectrometry (MS/MS) to identify both virus and host cell proteins.

Using a conservative false discovery rate (FDR) of less than 1%, 51 virus and 488 host cell proteins were identified in HSV-1 virions, and 51 virus and 883 host cell proteins were identified in BoHV-1 (Fig. 2B). When considering those proteins predicted to be conserved based on genome homology, the viral protein compositions of BoHV-1 and HSV-1 virions were broadly similar, with 42 virus proteins identified in virions of both viruses (Fig. 2C). Of the nine virus proteins that were uniquely identified in BoHV-1 virions, CIRC, U_S_1.67, and U_L_3.5 can be accounted for by a lack of conservation of these open reading frames within the HSV-1 genome. Likewise, five of the nine proteins uniquely identified in HSV-1 virions are not conserved in the BoHV-1 genome (U_L_45, U_L_55, U_L_56, U_S_10, and U_S_11). The full lists of virus proteins that were identified in HSV-1 and BoHV-1 virions are presented in Tables 1 and 2, respectively, organized according to their proposed localization within the virion (8, 10), with a side-by-side comparison provided in Table 3.

**TABLE 1:**
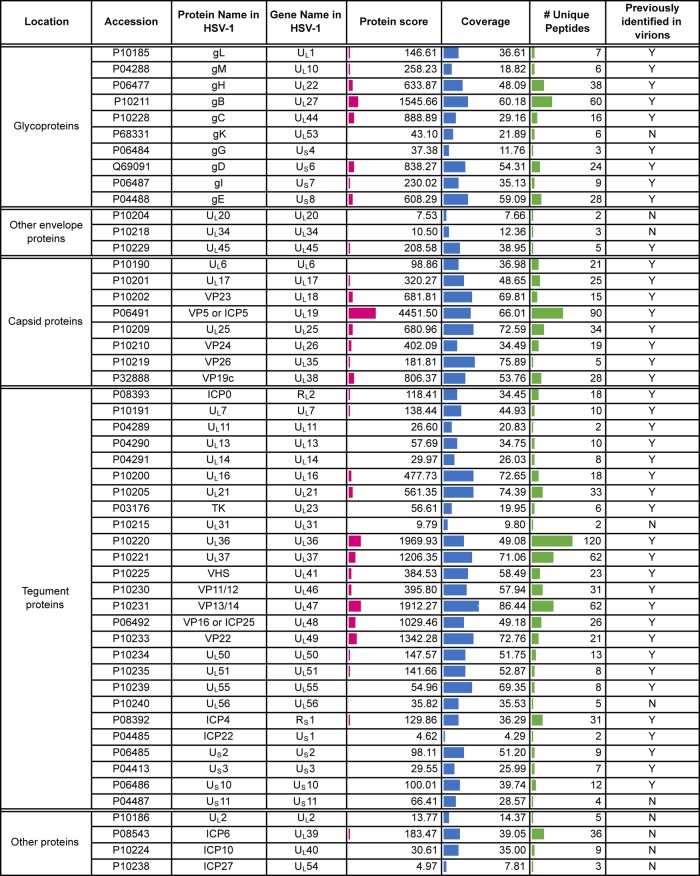
Virus proteins detected in extracellularHSV-1 virions^*a*^

a Shown are virus proteins identified based on a <1% FDR. Location is based on that described by Loret et al. (10), and detection in that previous study is indicated. The protein score reflects the number of peptides, abundance, and protein size to give a degree of confidence in protein identification. Coverage is calculated based on peptides identified within each protein. A side-by-side comparison of HSV-1 and BoHV-1 virions is presented in Table 3. Accession numbers were acquired from the UniProt human herpesvirus 1 (strain 17) database (https://www.uniprot.org/proteomes/UP000009294).

**TABLE 2:**
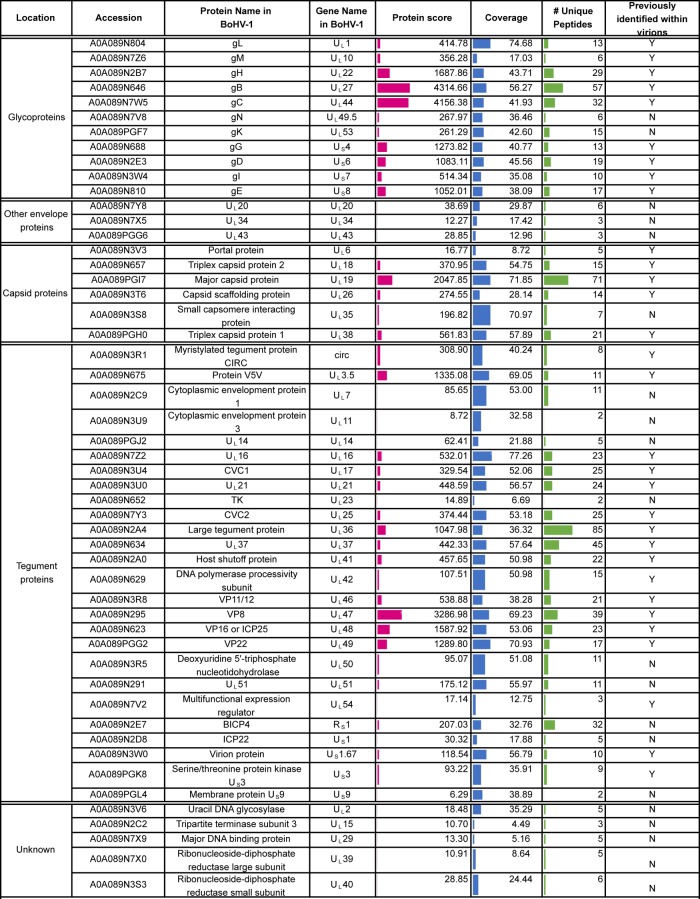
Virus proteins detected in extracellular BoHV-1 virions^*a*^

a Virus proteins were identified based on a <1% FDR. Location is based on that described by Barber et al. (8) or as expected based on the localization of homologous HSV-1 proteins, and detection in that previous study is indicated. The protein score reflects the number of peptides, abundance, and protein size to give a degree of confidence in protein identification. Coverage is calculated based on peptides identified within each protein. A side-by-side comparison of HSV-1 and BoHV-1 virions is presented in Table 3. Accession numbers were acquired from the UniProt bovine herpesvirus 1 (strain K22) database (https://www.uniprot.org/proteomes/UP000170085).

**TABLE 3:**
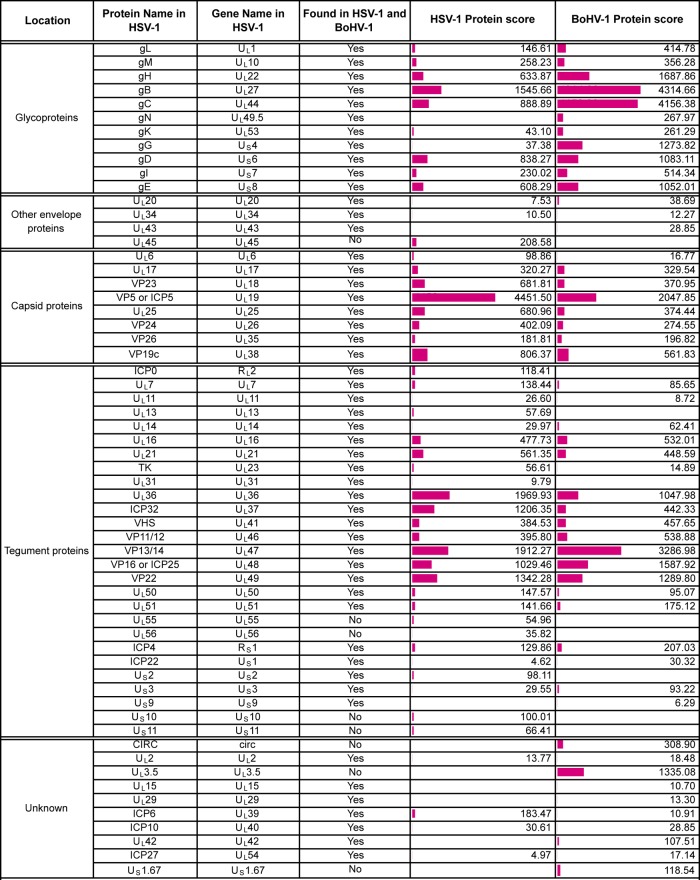
Comparison of BoHV-1 and HSV-1 virion compositions^*a*^

a Shown is a side-by-side comparison of BoHV-1 and HSV-1 virion compositions based on data presented in Tables 1 and 2 for HSV-1 and BoHV-1 homologues.

The viral content of HSV-1 extracellular virions was very similar to that previously reported by Loret and colleagues (10), confirming the presence of four novel virion components (U_L_7, U_L_23, U_L_50, and U_L_55) identified in that study, although we failed to detect ICP34.5, which has also been characterized elsewhere as a virion component (39). Likewise, the virus protein content of BoHV-1 virions was similar to that previously reported by Barber and colleagues (8). We also identified 20 proteins in BoHV-1 virions (Table 2) and 10 proteins in HSV-1 virions (Table 1) that were not identified in those original studies (8, 10). The low protein score and poor peptide coverage of these proteins suggest that they could be nonspecific, low-level contamination from cells that had been picked up due to the increased sensitivity of mass spectrometry. However, detection in virions from both the viruses and/or previous characterization of these proteins as virion constituents suggests that at least some of them, such as U_S_11 in HSV-1, are likely to be low-abundance constituents of the virion (40).

When considering those human host cell proteins for which there is a known bovine homologue, only a quarter of the host cell proteins identified was shared between the two viruses, variability which could be a consequence of isolating the virions from different cell types (Fig. 2D). A selection of the proteins that were identified in both BoHV-1 and HSV-1 virions is shown in Table 4, with the full list of host cell proteins identified in BoHV-1 and HSV-1 virions presented Tables S1 and S2 and a comparison presented in Table S3 in the supplemental material. While many of these proteins have been previously identified within herpesvirus virions (8, 10, 41), the specificity of their packaging remains to be determined. One potential explanation for the presence of so many cell proteins in our preparations is that exosomes have been purified along with our virions. While some exosomal markers (42) are present within our top cell protein hits (annexin A2, cofilin, heat shock protein 70, and GAPDH [glyceraldehyde-3-phosphate dehydrogenase]), it would be difficult to say with confidence that exosomes are present. Moreover, because many of these proteins are known to be among the most abundant proteins within the cell according to previously reported protein abundance data (43), another interpretation of their presence is that their recruitment is simply a reflection of local concentration at the site of envelopment rather than specific incorporation into the virion. In light of this uncertainty, we have not explored the packaging of cellular proteins further in this study.

**TABLE 4:**
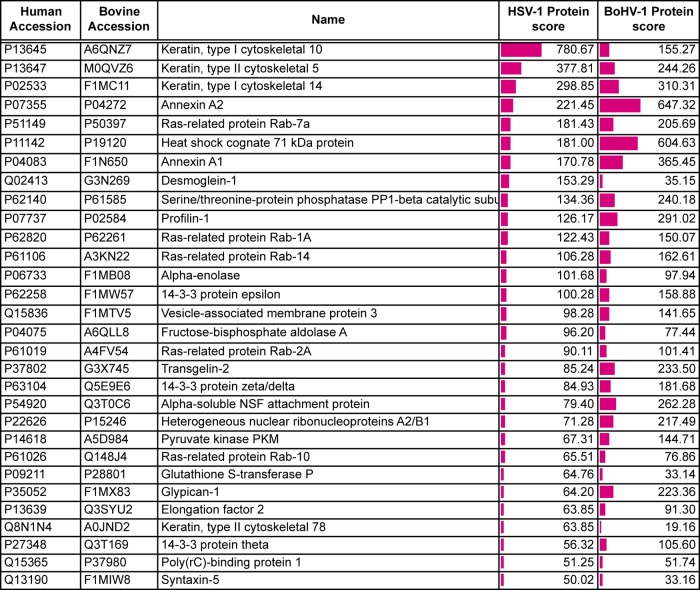
A subset of host cell proteins detected in extracellular HSV-1 and BoHV-1 virions^*a*^

a Shown are host cell proteins identified based on a <1% FDR. The protein score reflects the number of peptides, abundance, and protein size to give a degree of confidence in protein identification. Data sets are shown in full in Tables S1 to S3 in the supplemental material. Accession numbers were acquired from the UniProt human and *Bos taurus* databases (https://www.uniprot.org/proteomes/UP000005640 and https://www.uniprot.org/proteomes/UP000009136).

### A range of envelopment events in BoHV-1-infected cells.

**FIG 3:**
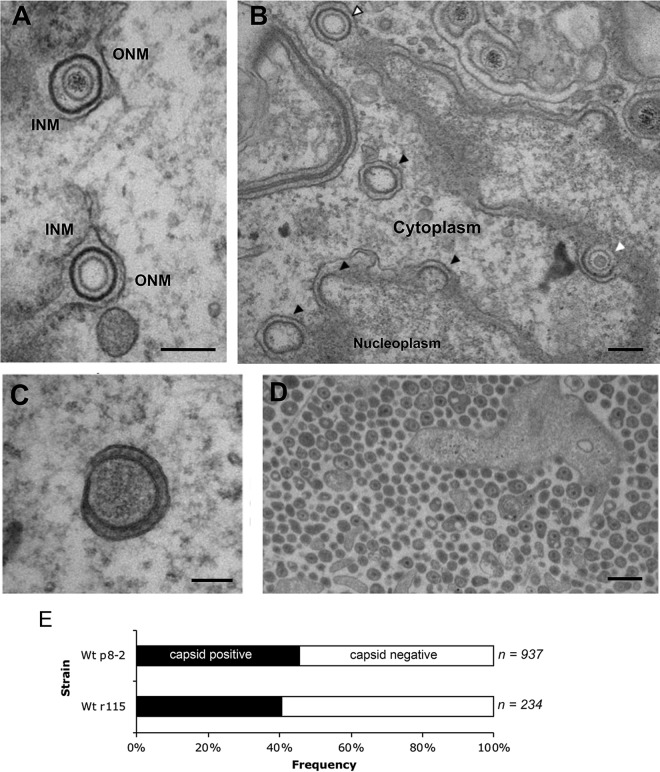
A variety of wrapping events occur in BoHV-1-infected cells. MDBK cells were infected at an MOI of 5 with BoHV-1 and fixed and processed for transmission electron microscopy at 12 hpi. (A and B) Representative sections of the nuclear envelope. In panel B, nuclear budding profiles that contain capsids resembling B capsids (white arrow) and A capsids (white arrow with black outline) or those occurring without a capsid (black arrows) are indicated. Bar = 200 nm. INM, inner nuclear membrane; ONM, outer nuclear membrane. (C) Fully wrapped capsidless L-particles were detected in the cytoplasm. Bar = 100 nm. (D) Multiple extracellular particles included full virions and capsidless L-particles. Bar = 500 nm. (E) Extracellular particles of MDBK cells infected with two strains of BoHV-1 were scored for the presence or absence of a capsid. Wt, wild type.

When examining BoHV-1-infected MDBK cells by standard transmission electron microscopy (TEM), we noted that these cells contained many more detectable primary envelopment events at the inner nuclear membrane (INM) than we had found in our previous studies of HSV-1-infected primary human fibroblasts (17). Assembled capsids surrounded by a distinct electron-dense primary envelope located in the perinuclear space between the INM and the outer nuclear membrane (ONM) were frequently observed (Fig. 3A). These primary virions either contained packaged DNA (Fig. 3A, top) or were empty of DNA (Fig. 3A, bottom). Three budding events lacking DNA were readily identified: budding of what appear to be B capsids (Fig. 3B, white arrow) and A capsids (Fig. 3B, white arrow with black outline) and membrane budding in the complete absence of a capsid (Fig. 3B, black arrows). Despite variations in cargo, each budding event involved similar INM curvatures, producing perinuclear vesicles of comparable diameters, and all events displayed an electron-dense coating on the inside surface of the curved membrane. These observations suggest that capsid association with the INM does not regulate budding of the INM in BoHV-1-infected cells and that, as is known for HSV-1 and PRV, scission of the budded INM is an autonomous process that can occur efficiently in the absence of a capsid cargo (16).

In addition to capsidless budding at the INM, we were also able to identify surprisingly large numbers of capsidless light particles (L-particles) within the cytoplasm (Fig. 3C). These particles were of the same diameter as full virions, were wrapped in a double membrane, and contained electron-dense material that is likely to be tegument (Fig. 3C). Moreover, the extracellular population of released particles contained a high proportion of capsidless L-particles (Fig. 3D), which when measured for two strains of BoHV-1 amounted to around 50% of all released particles (Fig. 3E). In comparison, we found approximately 2% capsidless L-particles in similar electron micrographs of HSV-1-infected cells (not shown).

### BoHV-1 particle assembly proceeds in the absence of DNA replication.

**FIG 4:**
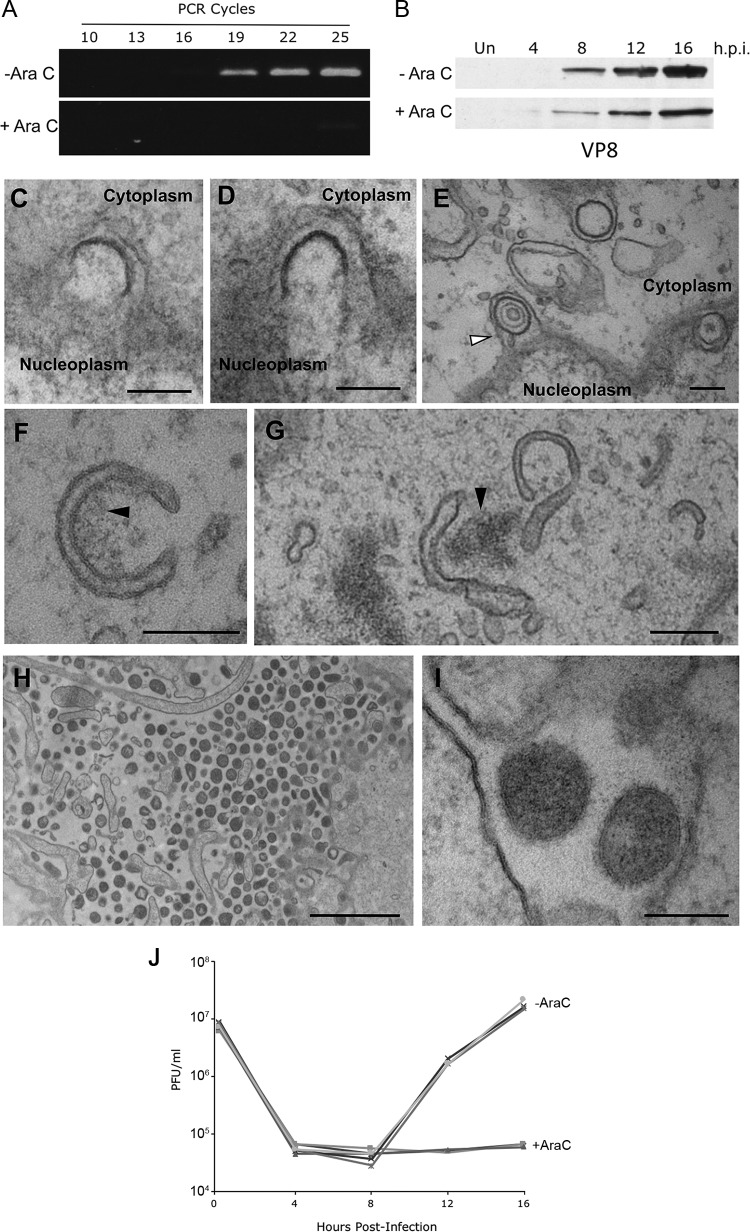
Inhibition of DNA replication with AraC does not arrest alternative wrapping events. (A and B) MDBK cells were infected at an MOI of 5 with BoHV-1 in the absence or presence of 100 μg/ml AraC. (A) The level of BoHV-1 genomic DNA was measured at 16 h by semiquantitative PCR using primers specific for U_L_47. (B) Synthesis of the late VP8 protein was measured by Western blotting of samples harvested at the indicated times. Un, uninfected. (C to I) MDBK cells were infected at an MOI of 5 with BoHV-1 in the presence of 100 μg/ml AraC. Samples were fixed and processed at 16 h, before processing for transmission electron microscopy. (C and D) Incomplete budding through the INM in the direction of the perinuclear space. (E) Several sealed vesicles are shown in the perinuclear space, with the arrow indicating a vesicle containing a B capsid. (F and G) Curved cytoplasmic tubules within the cytoplasm, frequently in association with electron-dense aggregates of material, indicated by the black arrows. (H and I) Extracellular space immediately adjacent to the plasma membrane, showing electron-dense particles, without a capsid cargo. Bars = 200 nm (C to G and I) and 2 μm (H). (J) MDBK cells were infected at an MOI of 5 with BoHV-1 in the absence (−AraC) or presence (+AraC) of 100 μg/ml AraC. Total virus was harvested at the indicated times and titrated on MDBK cells. Data from three biological replicates under each condition are shown.

Since both nuclear budding and particle egress can occur in the absence of a capsid cargo, we reasoned that, as for HSV-1 (24), both stages would be detectable in the absence of DNA replication. To examine this, BoHV-1 infection was perturbed by treatment with the nucleoside analogue arabinofuranosyl cytidine (AraC). AraC effectively blocked BoHV-1 genome replication as measured by semiquantitative PCR (Fig. 4A), while the late protein U_L_47 (VP8) was expressed albeit at lower levels than in untreated cells (Fig. 4B). TEM analysis of AraC-treated infected cells revealed a variety of budding events at the inner nuclear membrane, including incomplete budding through the INM (Fig. 4C and D) as well as fully formed perinuclear vesicles (Fig. 4E). A small number of these vesicles contained B capsids (Fig. 4E, white arrowhead), but the majority of them were empty, confirming that INM budding proceeds even when prior steps of virus assembly have not taken place.

In addition to nuclear budding, cytoplasmic membranes that had a degree of curvature similar to that observed in infection of untreated cells were identified (Fig. 4F and G). Although capsids were not associated with these membranes, their curvature and size were such that capsids could be accommodated. Electron-dense material was seen to be associated with some of these endocytic membranes, which may represent assembling tegument (Fig. 4F and G, black arrows). Densely packed clusters of capsidless particles with consistent diameters were also noted outside the infected cells (Fig. 4H), and these particles had apparent glycoprotein spikes on their surface (Fig. 4I). These electron micrographs indicate that particle production continued efficiently in the absence of BoHV-1 DNA replication, and we predict that these particles are L-particles enveloped via the normal envelopment route in the absence of capsid production. A one-step growth curve carried out in the absence or presence of AraC also confirmed that no infectious particles were produced under these conditions (Fig. 4J).

### Proteomic composition of L-particles generated during BoHV-1 and HSV-1 infection.

**FIG 5:**
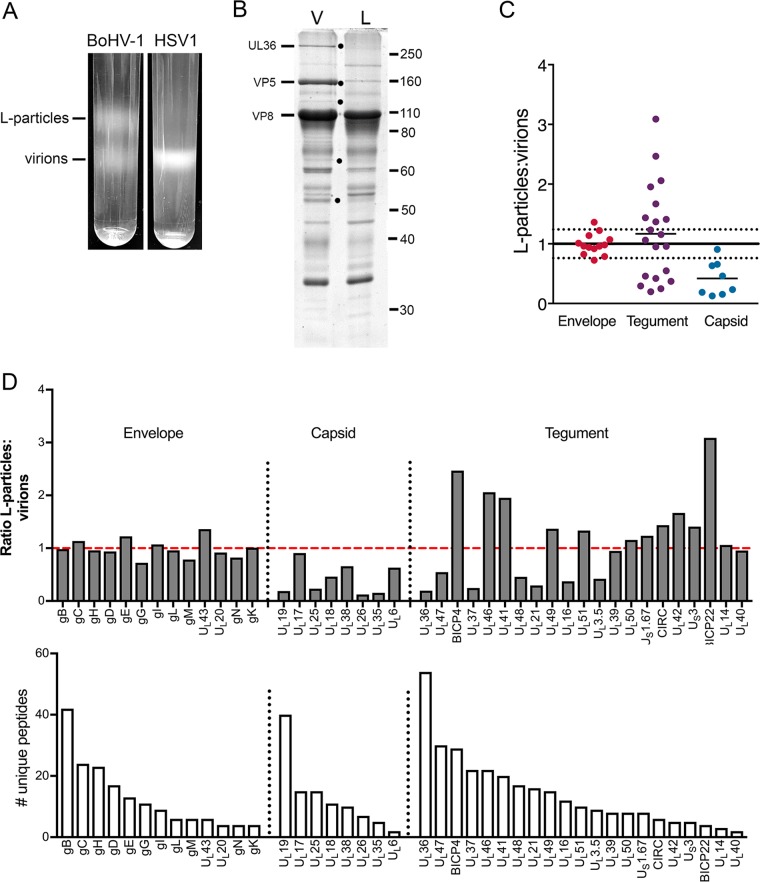
Quantitative proteomic comparison of the L-particles and virions produced during BoHV-1 infection. (A) Confluent monolayers of MDBK cells were infected with BoHV-1 strain P8-2, and HaCaT cells were infected with HSV-1 strain Sc16, both at a multiplicity of 0.02. At full cytopathic effect, the extracellular medium was harvested, and released particles were isolated on 5% to 15% Ficoll gradients. Representative Ficoll gradients of BoHV-1 and HSV-1 particle preparations isolated at the same time are shown. (B) Equal amounts of the virion (V) and L-particle (L) bands of BoHV-1 were separated by 10% SDS-PAGE and stained with Coomassie blue. Black dots denote protein bands present in virions but not L-particles. Size markers are shown in kilodaltons. (C) The BoHV-1 virion and L-particle samples were subjected to TMT labeling and analyzed by mass spectrometry. Shown is a summary of the normalized ratios of virus proteins detected in BoHV-1 L-particles to virions, grouped according to their predicted location within the virion. The normalization line represents the average of all glycoprotein ratios, with 1 standard deviation on either side represented by dotted lines. (D) Ratio of individual virus proteins in BoHV-1 L-particles compared to virions (top), in order of the number of unique peptides detected (bottom). The dashed red line is the glycoprotein normalization line.

To characterize the relative composition of BoHV-1 L-particles in comparison to virions, the upper (light) and lower (virion) bands on a Ficoll gradient (Fig. 5A) were harvested by needle puncture through the side of the tube and analyzed by SDS-PAGE, followed by staining with Coomassie blue (Fig. 5B). As before, bands corresponding to the sizes expected for several characteristic virus proteins were identified, with a band corresponding to the major capsid protein (VP5) detectable in virions but barely detectable in L-particles, suggesting negligible contamination of this sample with virions. Many of the proteins were present at similar levels in both samples, but at least five proteins were identified as being differentially packaged into virions, as indicated by black dots (Fig. 5B). While several of these are likely to be capsid proteins, at least one of them, U_L_36, is a known inner tegument protein. To compare the composition of L-particles to that of virions in greater detail, a duplex tandem mass tagging (TMT) system was used to label L-particle and virion samples, and mass spectrometry was used to identify and quantify the different proteins present in these samples (44). Initial analysis of the raw data indicated that, as expected, the L-particle/virion ratios of each virus glycoprotein were similar and ranged from 1 to 1.6 (Table 5). Therefore, to facilitate the subsequent analysis of the total data set, the light particle/virion ratios of all glycoproteins were averaged, and all light particle/virion ratios were normalized to this value. The results are summarized according to their predicted location in the envelope, tegument, or capsid (Fig. 5C), with total protein scores provided in Table 5. This shows that while envelope proteins were roughly equally abundant in the two populations, and the abundances of most of the proteins classified as capsid proteins were greatly reduced in L-particles, the BoHV-1 tegument proteins segregated into two groups: those that behaved like capsid proteins and were found mainly within virions and those that were equally as or more abundant within L-particles.

**TABLE 5:**
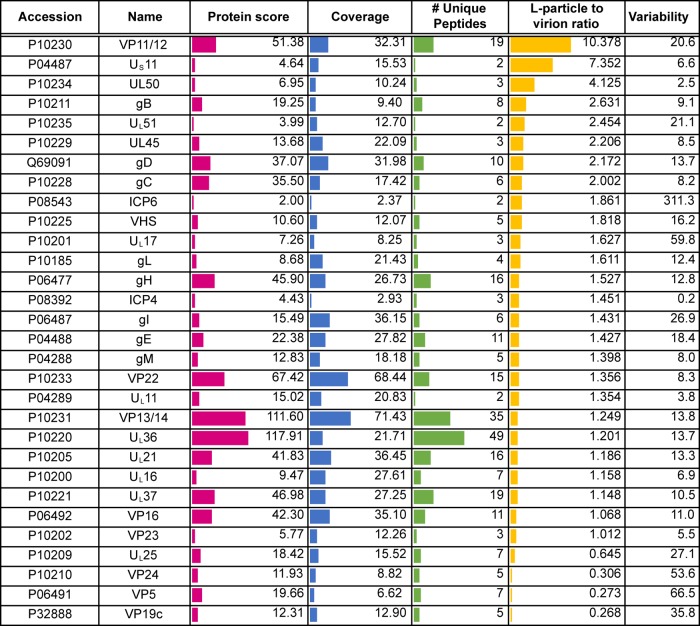
Results of TMT proteomic analysis of BoHV-1 L-particles and virions^*a*^

a BoHV-1 proteins were identified based on a <1% FDR, with the L-particle-to-virion ratio for each protein shown.

The ratios of individual proteins have been further grouped according to their predicted location within the virion and ranked according to the number of unique peptides identified for each of them (Fig. 5D). As also shown in Fig. 5C, the individual envelope proteins are tightly grouped around the normalization line (Fig. 5D). In contrast, the abundances of the capsid proteins U_L_19, U_L_25, U_L_26, and U_L_35 were greatly reduced in the L-particle samples (Fig. 5D). Interestingly, the U_L_17 protein was the most abundant capsid protein in the BoHV-1 L-particles, in agreement with a previous study showing U_L_17 as a component of both the tegument and capsid of HSV-1 (45). Among the tegument proteins, those that segregated with virions included the known inner tegument proteins U_L_36 and U_L_37 (Fig. 5D). Additionally, U_L_48, U_L_21, U_L_16, and U_L_3.5 proteins also appeared to be depleted from the L-particle samples. It should be noted that the results for those proteins to the right-hand side of each section of Fig. 5D (for example, the U_L_6 capsid protein and BICP22 in the tegument) should be treated with caution because of their low protein score and number of unique peptides identified (Fig. 5D). Nonetheless, the high protein scores for the more abundant tegument proteins provide confidence in the analysis, and taken together, this would suggest that each of the inner tegument proteins that is predicted to link the virus capsid to the tegument in the virion is barely present within our L-particle preparation. In short, BoHV-1 L-particles appear to be assembled without the inner tegument-capsid-interacting proteins.

**FIG 6:**
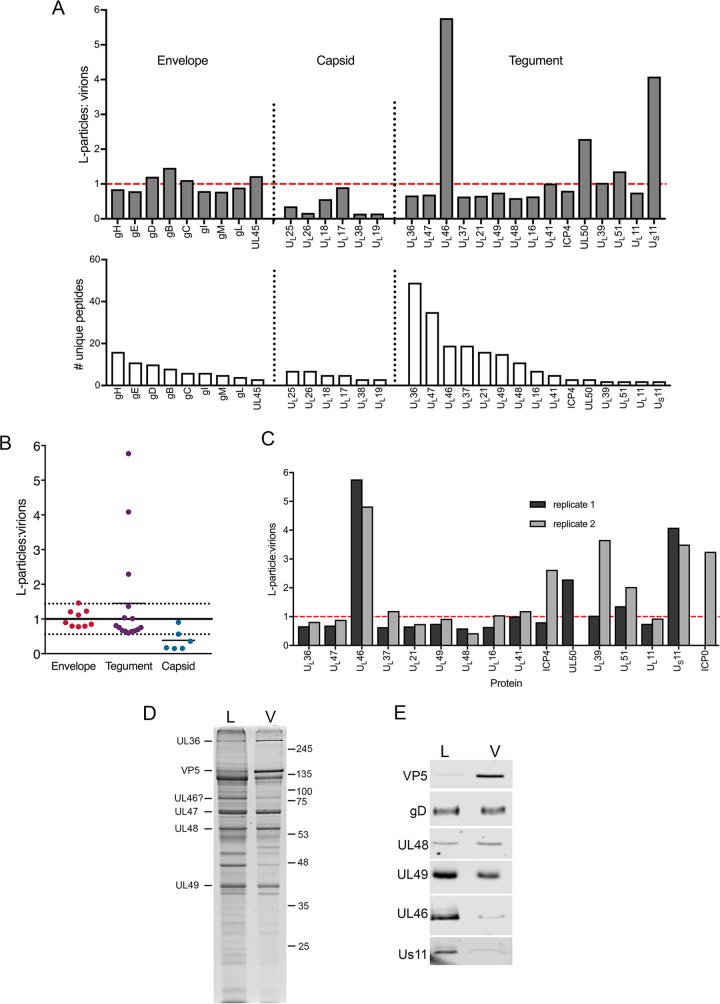
Quantitative proteomic comparison of the L-particles and virions produced in HSV-1 infection. (A) A Ficoll gradient for HSV-1 strain Sc16 similar to that shown in Fig. 5A was harvested for virions and L-particles as for BoHV-1 and analyzed by tandem mass tag labeling mass spectrometry as for BoHV-1. The results are presented as the ratio of the number of individual virus proteins in HSV-1 L-particles to virions (top), in order of the number of unique peptides detected (bottom). The dashed red line is the glycoprotein normalization line. (B) Summary of the normalized ratios of virus proteins detected in HSV-1 L-particles to virions, grouped according to their predicted location within the virion. The normalization line represents the average of all glycoprotein ratios, with 1 standard deviation on either side represented by dotted lines. (C) Comparative ratios of individual tegument proteins between two independent TMT labeling analyses. (D and E) Approximately equivalent amounts of Sc16 virions (V) and L-particles (L) were subjected to SDS-PAGE on a 10% polyacrylamide gel followed by Coomassie blue staining (D) or Western blotting (E) for the indicated virus proteins. Approximately equivalent numbers of particles were loaded by equating gD and U_L_48 by Western blotting. Molecular weight markers are shown in kilodaltons.

Although we have found that HSV-1 L-particles were much less abundant in extracellular purified particle populations from HaCaT cells than BoHV-1 from MDBK cells (Fig. 5A), we harvested the equivalent regions of the HSV-1 gradient as we had for the BoHV-1 gradient, which enabled us to isolate a small number of HSV-1 L-particles for proteomic analysis as described above for BoHV-1. In contrast to BoHV-1, most HSV-1 tegument proteins appeared to be largely equally abundant in virions and L-particles (Fig. 6A and B; total protein scores are provided in Table 6). In particular, further analysis revealed that unlike BoHV-1, the inner tegument proteins of HSV-1 were abundant in L-particles (Fig. 6A), as has been shown by others (22). Interestingly, U_L_46 (VP11/12) and U_S_11 were enriched around >6-fold and 4-fold, respectively, in L-particles, and U_L_50 was enriched to a lesser extent, >2-fold (Fig. 6A).

**TABLE 6:**
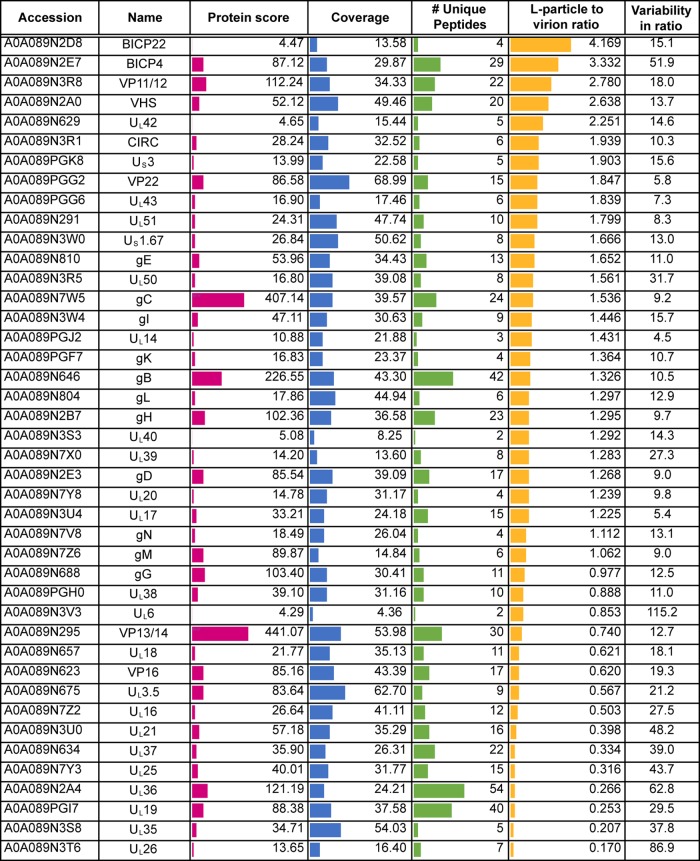
Results of TMT proteomic analysis of HSV-1 L-particles and virions^*a*^

a Shown are HSV-1 proteins identified based on a <1% FDR, with the L-particle-to-virion ratio for each protein shown.

Because the HSV-1 L-particles were difficult to isolate, and to ensure that this enhanced incorporation was reproducible, a second isolation of HSV-1 virions and L-particles was carried out and analyzed by TMT mass spectrometry. This confirmed that the tegument proteins were all approximately equally abundant in HSV-1 L-particles and virions, again with the exception of U_L_46 and U_S_11 (Fig. 6C). However, on this occasion, U_L_50 was undetectable in the samples, while ICP0 was detected in the second but not the first preparation. This showed that, as for BoHV-1, variability in the content of proteins detected by low peptide numbers could not provide confidence in the results to the extreme right-hand side of the graph. To further validate these proteomic results, approximately equivalent numbers of a third preparation of HSV-1 L-particles and virions, as judged by Coomassie blue staining (Fig. 6D), were analyzed by Western blotting for the presence of a range of virus proteins, as indicated (Fig. 6E). As predicted from the mass spectrometry analysis, gD, VP22, and VP16 were similarly abundant in L-particles and virions, while the major capsid protein VP5 encoded by U_L_19 was enriched in the virion sample. In contrast, both U_L_46 and U_S_11 were enriched in the L-particle sample, confirming the results of the mass spectrometry analysis (Fig. 6E). Of note, Western blotting of virions and L-particles for ICP34.5 failed to detect this protein despite a strong positive blot from infected cell lysates (data not shown). While we cannot exclude the possibility that the differences found in L-particle content between BoHV-1 and HSV1 were a consequence of the cell types used to purify the particles, these data highlight the fact that there are virus-specific variations in the makeup of noninfectious L-particles.

## DISCUSSION

In this study, we have extended our previous studies on HSV-1 morphogenesis (17) to provide evidence that BoHV-1 may follow a pathway similar to that of HSV-1. In ultrastructural studies, BoHV-1 wrapping events were similar to those previously found for HSV-1, with capsids associating with curved tubules which were positive for HRP that had been taken up by fluid-phase endocytosis. Given the growing evidence for the role of endocytosis at the molecular and ultrastructural levels for a range of herpesviruses (17, 20, 46–48), we propose that this model, which requires virus glycoproteins to first traffic to the plasma membrane before being retrieved into the endocytic membranes, may represent a unifying model of alphaherpesvirus envelopment. Many of the endocytic tubules found in the cytoplasm of BoHV-1-infected cells exhibited a reproducible membrane curvature and contained budded domains, with the diameter, density, and structure bearing strong similarities to those of endocytic clathrin coats previously reported in uninfected cells (49). The presence of clathrin-like coats has also been reported in cryo-electron microscopy (EM)-based studies of HSV-1 envelopment (17, 26). Similarly, our studies have shown that BoHV-1 exhibits the same primary envelopment profiles, budding at the INM and within the perinuclear space, that have been identified for several other herpesviruses, including HSV-1 and PRV (13). However, compared to our previous results with HSV-1, these profiles were numerous and readily detectable in BoHV-1-infected cells, indicating that these events occur either more frequently or more slowly than in HSV-1. In addition to ultrastructural studies, we have also carried out the first side-by-side proteomic study of HSV-1 and BoHV-1 virions, both of which have been previously characterized independently by mass spectrometry (8, 10). In general, our results are in agreement with those previous studies, despite differences in strains of virus and cell lines used to produce the extracellular virions and confirm that the complements of virus-encoded proteins packaged into virions are similar for both viruses. In the case of HSV-1, we confirmed the absence of glycoproteins U_L_43, gJ, and gN but were able to detect two glycoproteins, gK and U_L_45, that had not been previously identified in its proteome. In BoHV-1, we detected 20 more virion proteins than in the previous study, including several proteins that were also present in HSV-1 virions. Glycoprotein G, encoded by U_S_4, was found to be highly abundant in BoHV-1 virions but hardly detectable in HSV-1 virions. Nevertheless, it is important to consider than many of these constituents had mass spectrometry scores lower than that for the portal protein U_L_6, which is known to be present in just 12 copies in each virion. While protein score is not a direct readout of protein abundance in the sample, it is an indication that these proteins were difficult to detect in the virion samples. There are a number of potential explanations for this: (i) they are present in very low copy numbers in each particle, (ii) they are present in vastly variable numbers between particles, or (iii) they are simply contaminants from the cell extract that had been detected by the increased sensitivity of mass spectrometry. For example, the U_L_34 component of the NEC was detected at very low levels in particles from both viruses, but given its role at the nuclear membrane, it may not be expected to be incorporated into the mature particle. In addition, while the presence of glycoprotein K was convincing in both viruses, the level of its partner glycoprotein U_L_20 was extremely low (50, 51). Finally, despite the increased sensitivity of our mass spectrometry analysis, we failed to identify either ICP34.5 or U_S_9 in HSV-1 virions, both of which had been detected by only a single peptide in the previous proteomic study on HSV-1 (10).

We found that in BoHV-1-infected cells, the capsidless envelopment events occurred with great efficiency. As a consequence, we were able to isolate released L-particles and compare their composition to that of BoHV-1 virions by mass spectrometry. Intriguingly, this revealed that the BoHV-1 L-particles, although not retrieved as a completely homogeneous population free of contaminating virions, were depleted for U_L_36, U_L_37, and U_L_48, essential tegument proteins that link the capsid to the tegument/envelope structure of alphaherpesviruses (27). Three other tegument proteins, U_L_21, U_L_16, and U_L_3.5, were also notable for being depleted from the BoHV-1 L-particles. U_L_21 has been shown to be involved in nuclear egress of HSV-1 and HSV-2 capsids (52, 53), suggesting a potential role in bridging the capsid to the envelope. Likewise, U_L_16 has been shown to be required for the egress of HSV-2 capsids from the nucleus (54) but has not been designated this role in HSV-1, where deletion of U_L_16 has a limited effect on virus replication (55). Interestingly, a recent study from the Banfield group has shown that HSV-1 U_L_16 rescues the nuclear egress defect in the HSV-2 knockout virus, suggesting that HSV-1 has another factor absent from HSV-2 that fulfils this role in the absence of U_L_16 (56). In light of this, it is important to note that as for HSV-2, BoHV-1 U_L_16 is essential for BoHV-1 replication (57). Finally, the U_L_3.5 protein is not present in HSV-1, but for BoHV-1, it has been shown to interact with U_L_48 (58), an interaction which might also place it within the inner tegument. Taken together with the high abundance of these particles, and the fact that they were efficiently produced in the absence of DNA replication, we propose that BoHV-1 L-particles lack the inner tegument, represent enveloped outer tegument proteins, and are produced in an event that is autonomous and entirely independent of steps prior to final wrapping in the assembly pathway. In support of this, studies on other viruses have shown that L-particles are produced in the absence of DNA replication, DNA packaging, or delivery of capsids to the cytoplasm (24, 59, 60), indicating that at least some of the proteins that make up L-particles are sufficient to direct the envelopment process (22). Moreover, the fact that the complements of outer tegument proteins are similar in full virions and L-particles would suggest that they share the same morphogenesis and release pathway, in agreement with data from other studies on PRV (61).

In contrast, in our hands, HSV-1 L-particles were released from human keratinocytes in much lower numbers. While we recognize that the level of L-particle production appears to be cell type dependent for HSV-1 (62), our result in HaCaT cells is in agreement with our previous experience of isolating HSV-1 particles from both BHK21 and Vero cells, where we consistently recover large numbers of infectious virions but few observable L-particles, as illustrated in Fig. 5A. Unfortunately, it is not possible to compare the two viruses in the same cell line, as BoHV-1 is restricted in its host range, while, consistent with previous studies (63, 64), we have been unable to isolate HSV-1 particles from MDBK cells. Nonetheless, two replicate preparations of HSV-1 L-particles from HaCaT cells revealed that unlike BoHV-1, the inner tegument proteins were incorporated into HSV-1 L-particles to approximately the same level as in virions. Interestingly, the U_L_46 tegument protein was reproducibly enhanced around 5-fold in HSV-1 L-particles compared to virions, a feature that may provide clues to the HSV-1 L-particle assembly-and-trafficking pathway. In relation to this, the biological significance of L-particles remains unknown, but as suggested by others (65), it is tempting to speculate that BoHV-1 L-particles in particular could deliver large amounts of outer tegument proteins to uninfected cells ahead of or as an enhancement to infecting virus. As it is now becoming clear that many of these proteins may have roles in counteracting cellular antiviral responses (66), the initial infected cell could potentially send out a wave of these particles to either deliver proteins that can fight a preexisting antiviral state set up by interferons or prevent responses induced by subsequent incoming infection. It is therefore interesting to note that HSV-1 U_L_46 has recently been assigned a role in evading the STING-cGAS DNA-sensing pathway (67), while BoHV-1 U_L_47 is proposed to inhibit interferon signaling by binding to STAT1 (68).

In summary, the abundance and tractability of BoHV-1 L-particle production make this system particularly attractive to further our understanding of virus morphogenesis and the role of tegument proteins. Moreover, the potential therapeutic application of these noninfectious particles, such as vaccine development or the packaging and delivery of heterologous overexpressed proteins, is a much more realistic prospect given a system that is exquisitely designed to produce these particles efficiently and in large amounts.

## MATERIALS AND METHODS

### Cells and viruses.

Vero cells were cultured in Dulbecco's modified Eagle medium (DMEM) supplemented with 10% newborn calf serum (NCS) and 50 U/ml penicillin-streptomycin. HaCaT and MDBK cells were cultured in DMEM supplemented with 10% fetal bovine serum and 50 U/ml penicillin-streptomycin. HSV-1 strain Sc16 was routinely propagated and titrated in Vero cells in DMEM supplemented with 2% NCS and 50 U/ml penicillin-streptomycin. BoHV-1 strains P8-2 and r115 were routinely propagated and titrated in MDBK cells in DMEM supplemented with 2% NCS and 50 U/ml penicillin-streptomycin.

### Isolation of extracellular virus particles.

For mass spectrometry-based studies, released HSV-1 and BoHV-1 virions and L-particles were gradient purified as described previously (69). Briefly, 10 175-cm^2^ flasks of confluent MDBK or HaCaT cells (approximately 6 × 10^8^ cells in total) were infected with BoHV-1 (strain P8-2) or HSV-1 (strain Sc16) at a multiplicity of 0.02. Once cytopathic effect was advanced (3 to 4 days postinfection), the extracellular medium was collected and centrifuged at 3,000 rpm for 30 min at 4°C in a fixed-angle rotor to remove cell debris. Virus particles were then pelleted from the cleared supernatant at 9,000 rpm for 90 min at 4°C. The particle pellet was resuspended in 0.5 ml phosphate-buffered saline (PBS) and carefully layered onto a preformed 11-ml 5% to 15% (wt/vol) Ficoll gradient in a 13.2-ml thin-wall polyallomer ultracentrifuge tube (Beckman Coulter). Gradients were centrifuged at 12,000 rpm for 2 h at 4°C in an SW41 Ti swinging-bucket rotor in a Sorvall Discovery SE ultracentrifuge. Light and heavy bands were harvested by needle puncture through the side of the tube with a 19-gauge hypodermic needle in a volume of <1 ml, diluted in 10 ml PBS, and pelleted at 25,000 rpm for 1 h at 4°C using the same rotor and ultracentrifuge. The pellets were resuspended in a suitable volume of PBS and stored at −80°C.

### SDS-PAGE and Western blotting.

Virus particle samples were separated by 9 or 10% SDS-PAGE and stained with Coomassie blue or transferred to nitrocellulose membranes before Western blotting. The following primary antibodies were used for Western blots and kindly provided: mouse anti-gD (LP14), from Tony Minson (University of Cambridge); mouse anti-VP16 (LP1), from Colin Crump (University of Cambridge); rabbit anti-U_L_46, from Richard Courtney (Pennsylvania State University); rabbit anti-U_S_11, from Ian Mohr (New York University); and mouse anti-VP5 (HA-018; Virusys). Our rabbit VP22-specific antibody (AGV031) has been described previously (70, 71). Goat anti-mouse IRDye 680RD and goat anti-rabbit IRDye 800CW (Li-Cor Biosciences) secondary antibodies were used as appropriate, before blots were imaged using an Odyssey CLx imaging system (Li-Cor Biosciences).

### Conventional proteomic analysis of BoHV-1 and HSV-1 virions.

BoHV-1 or HSV-1 virion samples were separated by SDS-PAGE, the gel lane was cut into three slices, and each slice was subjected to in-gel tryptic digestion using a DigestPro automated digestion unit (Intavis). The resulting peptides were fractionated using a nano-HPLC system with an LTQ-Orbitrap Velos mass spectrometer (ThermoFisher Scientific).

### TMT proteomic analysis of BoHV-1 and HSV-1 L-particles and virions.

A total of 100 μg of protein was digested with 2.5 μg trypsin overnight at 37°C and labeled with tandem mass tag (TMT) sixplex reagents according to the manufacturer's protocol (ThermoFisher Scientific). The virion and L-particle samples were then pooled, evaporated to dryness, resuspended in 5% (vol/vol) formic acid, and then desalted using Sep-Pak cartridges according to the manufacturer's instructions (Waters). The eluent from the Sep-Pak cartridge was evaporated to dryness and resuspended in 1% (vol/vol) formic acid before analysis by the nano-HPLC system with an Orbitrap Fusion Tribrid mass spectrometer (ThermoFisher Scientific).

### Nano-HPLC mass spectrometry.

Samples were fractionated using an Ultimate 3000 nano-HPLC system. In brief, peptides in 1% (vol/vol) formic acid were injected onto an Acclaim PepMap C_18_ nano-trap column (ThermoFisher Scientific). After washing with 0.5% (vol/vol) acetonitrile–0.1% (vol/vol) formic acid, peptides were resolved on a 250-mm by 75-μm Acclaim PepMap C_18_ reverse-phase analytical column (ThermoFisher Scientific) over a 150-min organic gradient, using seven gradient samples for conventional samples (1 to 6% solvent B [aqueous 80% {vol/vol} acetonitrile in 0.1% {vol/vol} formic acid] over 1 min, 6 to 15% solvent B over 58 min, 15 to 32% solvent B over 58 min, 32 to 40% solvent B over 5 min, and 40 to 90% solvent B for 6 min, which was then reduced to 1% solvent B over 1 min) or six gradient segments for TMT-labeled samples (5 to 9% solvent B over 2 min, 9 to 25% solvent B over 94 min, 25 to 60% solvent B over 23 min, and 60 to 90% solvent B over 5 min, which was held at 90% solvent B for 5 min and then reduced to 1% solvent B over 2 min), with a flow rate of 300 nl min^−1^. Peptides were ionized by nano-electrospray ionization at 2.0 or 2.1 kV using a stainless steel emitter with an internal diameter of 30 μm (ThermoFisher Scientific) and a capillary temperature of 275°C.

For conventional proteomics, tandem mass spectra were acquired using an LTQ-Orbitrap Velos mass spectrometer controlled by Xcalibur 2.1 software (ThermoFisher Scientific) and operated in the data-dependent acquisition mode. The Orbitrap was set to analyze the survey scans at a 60,000 resolution (at *m/z* 400) in the mass range *m/z* 300 to 2,000, and the top 20 multiply charged ions in each duty cycle were selected for tandem mass spectrometry (MS/MS) in the LTQ linear ion trap. Charge-state filtering, where unassigned precursor ions were not selected for fragmentation, and dynamic exclusion (repeat count, 1; repeat duration, 30 s; exclusion list size, 500) were used. Fragmentation conditions in the LTQ instrument were as follows: normalized collision energy of 40%, activation *q* of 0.25, activation time of 10 ms, and minimum ion selection intensity of 500 counts.

For TMT-labeled samples, all spectra were acquired by using an Orbitrap Fusion Tribrid mass spectrometer controlled by Xcalibur 2.0 software (ThermoFisher Scientific) and operated in the data-dependent acquisition mode using an SPS-MS3 workflow. FTMS1 spectra were collected at a resolution of 120,000, with an automatic gain control target of 400,000 and a maximum injection time of 100 ms. Precursors were filtered with an intensity range from 5,000 to 1 × 10^20^, according to charge state (to include charge states 2 to 6), and with monoisotopic precursor selection. Previously interrogated precursors were excluded using a dynamic window (60 s ± 10 ppm). The MS2 precursors were isolated with a quadrupole mass filter set to a width of 1.2 *m/z*. ITMS2 spectra were collected with an AGC target of 10,000, a maximum injection time of 70 ms, and CID collision energy of 35%.

For FTMS3 analysis, the Orbitrap was operated at a 30,000 resolution with an AGC target of 50,000 and a maximum injection time of 105 ms. Precursors were fragmented by high-energy collision dissociation at a normalized collision energy of 55% to measure maximal TMT reporter ion yield. Synchronous precursor selection was enabled to include up to five MS2 fragment ions in the FTMS3 scan.

### Proteomic data analysis.

The raw data files were processed and quantified using Proteome Discoverer software v1.4 (ThermoFisher Scientific). The data were searched against either the UniProt human database (downloaded on 18 April 2016; 134,169 entries) and the UniProt human herpesvirus 1 (strain 17) database (downloaded on 11 April 2016; 73 entries) or the UniProt Bos taurus database (downloaded on 21 October 2016; 31,855 entries) and the UniProt bovine herpesvirus 1 (strain K22) database (downloaded on 21 October 2016; 69 entries) as appropriate. All searches were performed using the SEQUEST algorithm. The peptide precursor mass tolerance was set at 10 ppm, and the MS/MS tolerance was set at 0.8 or 0.6 Da. Search criteria included oxidation of methionine (+15.9949) as a variable modification and carbamidomethylation of cysteine (+57.0214) and the addition of the TMT mass tag (+229.163) to peptide N termini and lysine as fixed modifications. Searches were performed with full tryptic digestion, and a maximum of one missed cleavage was allowed. The reverse database search option was enabled, and all peptide data were filtered to satisfy a false discovery rate (FDR) of 5% or 1%.

### Transmission electron microscopy.

To prepare samples for electron microscopy, cells were grown to confluence overnight before infection with virus. When infection was carried out in the presence of AraC, all infection, washing, and incubation steps were performed in the presence of 100 ng/ml AraC. Horseradish peroxidase (HRP) labeling was performed as previously described (17), by incubating cells with 10 mg/ml HRP for 30 min prior to fixation. After fixation, samples were washed and stained with a metal-enhanced 3,3′-diaminobenzidine (DAB) substrate kit (ThermoFisher Scientific). The samples for electron microscopy were fixed and processed as previously described (72).

## Supplementary Material

Supplemental file 1
